# Implementing out of hours MDT safety huddles at the Ladywell Unit, Lewisham, South London and the Maudsley (SLAM) NHS Foundation Trust

**DOI:** 10.1192/bjo.2021.612

**Published:** 2021-06-18

**Authors:** Su Yeoh, Natavan Babayeva, Hugh Williams, Emma Jones

**Affiliations:** Ladywell unit, Lewisham, SLAM

## Abstract

**Aims:**

- For 100% of patients admitted OOH (Friday 5pm – Sunday 9am) to have a multi-disciplinary review of their treatment
- For 100% of patients with deteriorating physical and mental health to be discussed
- To improve multi-disciplinary team (MDT) morale, working relationships and team cohesiveness OOH

**Background:**

In most specialties, the standard of best practice is that patients admitted to the ward out of hours (OOH) receive a senior review over the weekend. However this does not usually take place in Psychiatry, and patients routinely wait until Monday to be seen by the ward team. This has been highlighted as problematic in cases where patients are agitated and not receiving any treatment for > 24 hours.

We trialled a weekend teleconference safety huddle in Lewisham involving the on call consultant, registrar (SpR), core trainee (CT) and duty senior nurse (DSN).

**Method:**

The weekend huddles were through a teleconference line, with participants dialling in at 9.45am.

Issues discussed:

1. Management plan for newly admitted patients OOH.
2. Plan for patients with deteriorating mental health or escalating level of aggression.
3. Plan for patients with deteriorating physical health.

Feedback was collected from CTs, SpRs and consultants focusing on whether the huddle made any difference to the speed of care, cohesiveness of the OOH team, and whether it was generally helpful or not.

**Result:**

54% of CTs (n = 11) felt that patients admitted OOH had an MDT review, and 90% felt that patients with deteriorating mental and physical health were discussed and a plan put in place. 80% of CTs, 63% of SpRs (n = 8) and 67% of consultants (n = 6) agreed it improved team cohesiveness. 90% of DSNs felt safer and more supported in decision-making OOH.

80% of CTs, 63% of SpRs and 83% of consultants found weekend huddles helpful.

Data were also collected on violent incidents OOH, and there was a slight reduction in the number of violent incidents in the weeks following introduction of the huddle.

**Conclusion:**

Introducing safety huddles in Lewisham has facilitated the prompt discussion of the management of patients admitted OOH, and of those with deteriorating mental and physical health. It has also fostered a greater sense of cohesiveness in the MDT team.

In light of this feedback, safety huddles have now been established as part of the weekend schedule in Lewisham, and are being rolled out to other boroughs within SLaM.

